# Congenital Zika virus syndrome…what else? Two case reports of severe combined fetal pathologies

**DOI:** 10.1186/s12884-018-1998-4

**Published:** 2018-09-03

**Authors:** Manon Vouga, David Baud, Eugénie Jolivet, Fatiha Najioullah, Alice Monthieux, Bruno Schaub

**Affiliations:** 1 Materno-Fetal and Obstetrics Research Unit, Department « Femme-Mère-Enfant », University Hospital, Lausanne, Switzerland; 2 Multidisciplinary Center of Prenatal Diagnosis of Martinique, Unit of Obstetrics and Gynecology, Maison de la Femme de la Mère et de l’Enfant, University Hospital of Martinique, Fort de France, France; 3 Registre des Malformations des Antilles (REMALAN), Maison de la Femme de la Mère et de l’Enfant, University Hospital of Martinique, Fort de France, France; 4 Service de Virologie, University Hospital of Martinique, Fort de France, France; 5 Materno-fetal & Obstetrics Research Unit, Department of Obstetrics and Gynecology, Centre Hospitalier Universitaire Vaudois (CHUV), 1011 Lausanne, Switzerland

**Keywords:** Congenital Zika syndrome, Parvovirus B19, Trisomy 18, Microcephaly, Fetal anemia

## Abstract

**Background:**

Zika virus (ZIKV) has recently emerged as a teratogenic infectious agent associated with severe fetal cerebral anomalies. Other microorganisms (TORCH agents) as well as genetic disorders and toxic agents may lead to similar anomalies. In case of fetal anomalies, the exact etiology might be difficult to establish, especially in ZIKV endemic countries. As the risks associated with maternal infection remain unclear adequate parental counseling is difficult.

**Case presentation:**

We present two cases of severe fetal pathologies managed in our multidisciplinary center during the ZIKV outbreak in Martinique, a French Caribbean Island. Both fetuses had congenital ZIKV infection confirmed by RT-PCR. While one case presented with significant cerebral anomalies, the other one presented with hydrops fetalis. A complete analysis revealed that the fetal lesions observed resulted from a combination of ZIKV congenital infection and a genetic disorder (trisomy 18) in case 1 or congenital Parvovirus B19 infection in case 2.

**Conclusions:**

We highlight the difficulties related to adequate diagnosis in case of suspected ZIKV congenital syndrome. Additional factors may contribute to or cause fetal pathology, even in the presence of a confirmed ZIKV fetal infection. An exact diagnosis is mandatory to draw definitive conclusions. We further emphasize that, similarly to other congenital infections, it is very likely that not all infected fetuses will become symptomatic.

## Background

2016 has seen the emergence of Zika virus (ZIKV), a member of the *Flaviviridae* family, as a teratogenic agent associated with severe fetal malformations. Several other microorganisms, grouped under the TORCH acronym (Toxoplasmosis, Others [Syphilis, Parvovirus B19, Varicella zoster, HIV], Rubella, Cytomegalovirus, Herpes), as well as genetic disorders and toxic agents may lead to similar anomalies [1].

Martinique is a French Caribbean Island that was affected by the ZIKV epidemic at the end of 2015, and presented one of the largest incidence rate among the Caribbean, with an estimated 36,590 potential cases during the epidemic [2]. To overcome the unknown risks of fetal anomalies associated with maternal infection, the French government implemented an active surveillance of all pregnant women during the epidemic, with monthly ultrasound examinations. In France, prenatal screening for aneuploidy and serological screening are offered to all pregnant patients during the first trimester. Screening for congenital infections (i.e. TORCH screening) is proposed in cases of suspected maternal/fetal infection. Blood was stored to allow retrospective serologic analyses. We present here two cases of severe fetal pathologies resulting from a combination of ZIKV congenital infection and either a genetic disorder or co-infection with Parvovirus B19, monitored in our reference center, and highlight the need to obtain an exact diagnosis in case of suspected ZIKV congenital syndrome.

## Case presentation

### Case 1

**Fig. 1:**
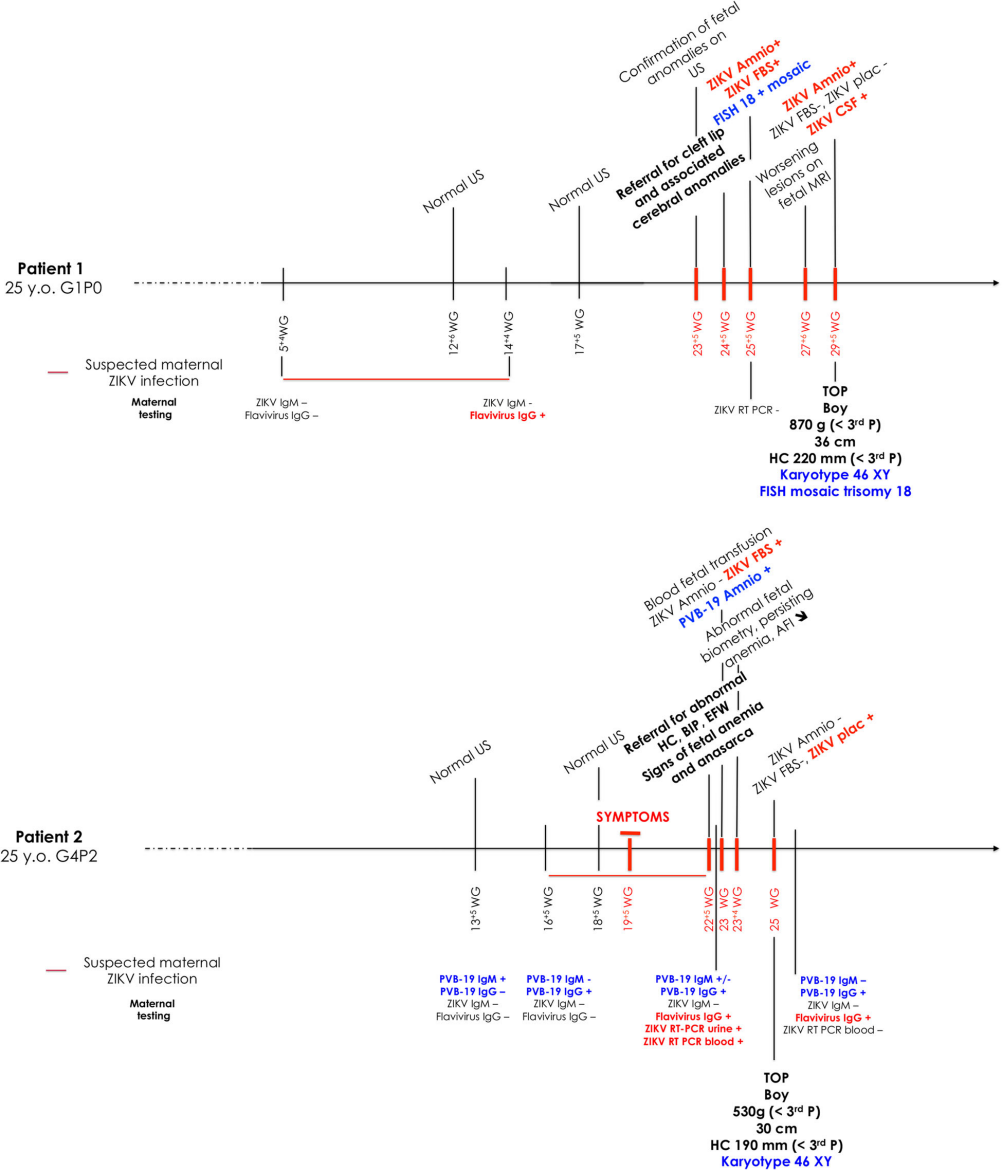
Monitoring over time. This figure represents clinical monitoring of two cases over time as well as specific outcomes. Abbreviations: Amnio, Amniocentesis; FBS, Fetal blood sampling; Plac, Placenta; PVB-19, Parvovirus B19; ZIKV, Zika virus

**Fig. 2:**
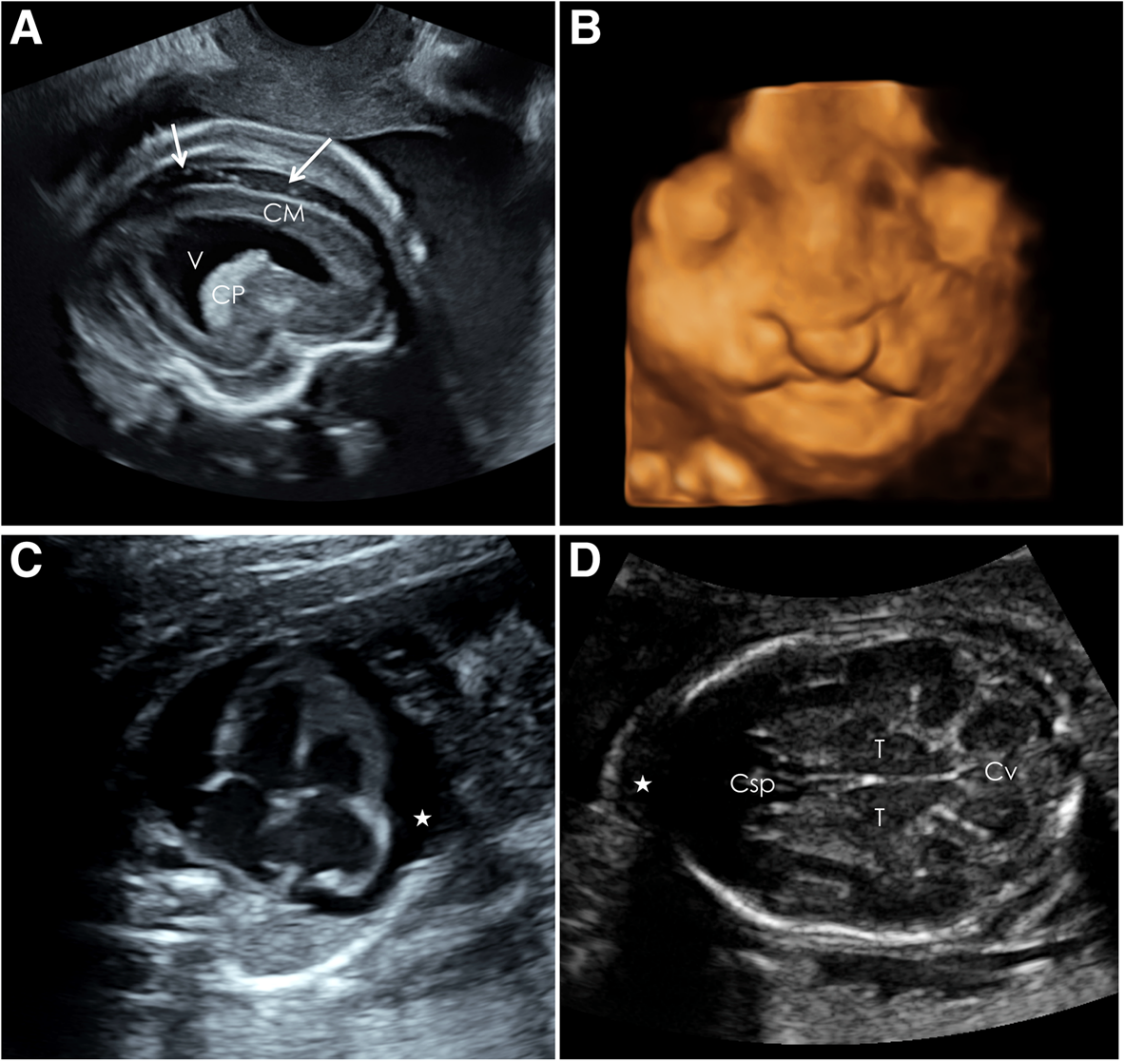
Specific ultrasound findings. This figure presents illustrative ultrasound scan images of Case 1 (**a** & **b**) and Case 2 (**c** & **d**). **a** Parasagittal plane at 24 + 5 WG showing the dilatation of the posterior horn of the lateral ventricle (V), the atrophy of the cortical mantle (CM), as well as enlarged peri-cerebral spaces, marked by an arrow. CP, choroid plexus. **b** Bilateral cleft palate observed at 27 + 6 WG on 3D reconstruction ultrasound scan. **c** Axial plane at 22 + 5 WG showing normal heart with a diffuse pericardial effusion (*). **d** Transcerebellar axial plane at 23 + 4 WG showing normal brain structures. Csp, Cavum spetum pellucidum; Cv, Cerebellar vermis, T, Thalami. Note the prefrontal edema (*)

Patient 1 was a healthy 25-year-old primigravida woman without any familial medical history. She experienced an uneventful pregnancy until 23 + 5 weeks gestation (WG), when the anatomical scan demonstrated a potential cleft lip associated with dilatation of the left cerebral ventricle and a short corpus callosum. The patient did not recall any particular illness previously in her pregnancy (see the Timeline presented in Fig. 1). The scan performed at 24 + 5 WG in our unit identified intra-uterine growth restriction (IUGR; <5th percentile) concurrent with multiple fetal anomalies including a bilateral cleft palate, colpocephaly (both atriums measuring 10 mm), dysgenesis of the corpus callosum and enlargement of the cisterna magna (14 mm) associated with a posterior fossa cyst (Fig. 2a and b). A diagnostic amniocentesis and fetal blood sampling were performed at 25 + 5 WG for congenital infections and genetic analysis. Toxoplasmosis, rubella and syphilis were previously excluded by negative maternal serology. Due to the ongoing ZIKV epidemic, ZIKV screening (RT-PCR RealStar® ZIKV RT-PCR Kit 1.0, Altona Diagnostics, Hamburg, Germany) was added to the routine testing despite the absence of suggestive symptoms in the mother. The amniotic fluid and fetal blood were positive for ZIKV RNA confirming a congenital ZIKV infection. Fetal biological parameters (Table 1) also suggested fetal infection with elevated total IgM (120 [<20 mg/l]); β2-Microglobulin 4.8 [<5 mg/ml]) and a cholestasis-like pattern (GGT 589 [<100 UI/l], AST 17 [<20 UI/l]), associated with moderate anemia and thrombocytopenia (Hb 13.4 [>15 g/100 ml], platelets 128 [>150 G/L]). However, maternal blood and urine were negative for ZIKV RNA. We therefore performed retrospective serum analysis, which confirmed a maternal ZIKV infection that occurred between 5 + 3 and 14 + 3 WG (see Fig. 1). Fetal serological analyses were negative for ZIKV IgM and equivocal for IgG, which may have resulted from materno-fetal passage. Meanwhile, an initial FISH analysis revealed mosaic trisomy 18 with 36% of cells affected.

**Table 1:** Biochemical and hematological fetal parameters

	Normal ranges	Case 1		Case 2	
		25 + 5 WG	29 + 5 WG	23 WG	25 WG
General parameters					
Total IgM [mg/l]	<20	120	70	ud.	49
β2-Microglobulin [mg/ml]	<5	4.8	4.2	ud.	7.8
Liver function					
GGT [UI/l]	<100	589	672	ud.	12
AST [UI/l]	<20	17	24	ud.	96
Albumin [g/l]	20–35	25.3	24.8	ud.	6.8
Total protein [g/l]	20–30	34	36	ud.	13
Hematological parameters					
Hb [g/100 ml]	>15	13.4	13.5	3.6	2.4
Platelets [G/L]	>150	128	149	68	17

*Abbreviation*: *ud.* undetermined, WG = Weeks Gestation

Due to the poor prognosis, the patient opted for termination of pregnancy as permitted by French legislation. At 29 + 5 WG, a stillborn male infant was delivered with a weight of 870 g (<3rd percentile), head circumference (HC) of 220 mm (<3rd percentile) and length of 36 cm, presenting with dysmorphic features: a large bilateral cleft lip, peaked palate, exophthalmia and hyperthelorism. At the time of delivery, fetal blood was negative for ZIKV RNA, however it was amplified from the cerebro-spinal fluid confirming ZIKV persistence in the central nervous system despite systemic clearance. Ultimately, cell culture and complete cytogenetic analysis confirmed the diagnostic of mosaic trisomy 18, while CGH array did not reveal any additional genetic anomalies. Autopsy was declined.

### Case 2

Patient 2 was a 25-year-old healthy, gravida 4 para 2, woman with a past history of stillbirth at 7 months gestation of unknown etiology. Screening test for aneuploidy was at low risk (1/10,000). At 19 + 5 WG, the patient experienced a maculo-papular pruritic rash associated with mild fever, arthralgia and headaches, compatible with a viral infection. The anatomical scan at 22 + 5 WG showed hydrops fetalis with skin edema, ascites, a large pericardial effusion and prefrontal edema (5.9 mm), associated with symmetric IUGR (estimated fetal weight 427 g; <3rd percentile, biparietal diameter (BPD) of 44 mm (<3rd percentile), HC of 165 mm (<3rd percentile), abdominal circumference of 193 mm (measurement unreliable secondary to presence of ascites), and femur length of 29.7 mm (<3rd percentile). No morphological anomalies were detected (Fig. 2c and d). Non-alloimmune fetal anemia was highly suspected. Amniocentesis and fetal blood sampling were performed, which confirmed severe fetal anemia and thrombocytopenia (Table 1) (Hb 3.6 [>15 g/100 ml]; platelets 68 [>150 G/L]). Fetal blood transfusion was performed. The amniotic fluid was positive for PVB-19 DNA, while negative for ZIKV RNA and CMV DNA. However, ZIKV RNA was detected in fetal blood and placenta confirming a combined PVB-19 and ZIKV fetal infection. Congruently, ZIKV RNA was detected in both maternal blood and urine. As persistent viremia has been described in pregnant women [3], timing of maternal infection was confirmed based on retrospective serum analysis. The identification of PVB-19 specific IgM at 13 + 5 WG, while both ZIKV specific IgG and IgM were negative confirmed a primary PVB-19 infection in the first trimester (see Timeline presented in Fig. 1). Maternal ZIKV infection occurred later and probably correlated with maternal symptoms at 19 + 5 WG. These unspecific symptoms could have corresponded to either a primary ZIKV or PVB-19 infection. Fetal blood analysis revealed an elevation of general inflammatory markers (β2-Microglobulin 7.8 [mg/ml] (<5), total IgM 49 [mg/l] (<20)). Unlike case 1, the GGT remained low, while hepatic cytolysis was observed (GGT 12 [UI/l] (<100), AST 96 [UI/l] (<20)). Due to worsening fetal clinical status after transfusion (absent end diastolic flow on umbilical artery Doppler, increasing ascites, pericardial effusion and skin edema), the patient requested medical termination of pregnancy, which was performed at 25 WG. A stillborn male infant weighing 530 g (<3rd percentile) presenting with significant edema was delivered. No other macroscopic anomalies were noted. Autopsy was declined.

## Discussions and conclusions

We present two cases of severe fetal pathology. Although fetal ZIKV infection was confirmed in both cases, it is unclear whether the fetuses were symptomatic secondary to ZIKV infection or their other pathology. We clearly showed that if Zika testing is positive, but the fetal pathology does not clearly fit congenital ZIKV syndrome, more investigations are needed. Both trisomy 18 and ZIKV are known to cause microcephaly, IUGR, colpocephaly and dysgenesis of the corpus callosum as observed in case 1 [4, 5]. Cleft palate has been described as part as the Pierre-Robin-sequence in two fetuses exposed to ZIKV during the French Polynesian outbreak, though neither maternal nor fetal infections were confirmed [6]. This finding is probably unrelated to ZIKV infection, which seems to have specific neurotropism. Interestingly, fetus 2 did not present with any cerebral anomalies. Though structures were small for GA, in accordance with the symmetric IUGR, no additional anomalies were seen. In particular, calcifications, ventriculomegaly and agenesis of the corpus callosum, which have been commonly described in ZIKV infected fetuses, were not observed [5]. Although two cases [5, 7] have been reported with hydrops fetalis in association with ZIKV fetal infection, it does not seem to be a common finding. Furthermore, severe anemia and thrombocytopenia have not been described in association with ZIKV infection [3]. This suggests that PVB-19 infection was primarily responsible for the symptoms in fetus 2. The proximity of ZIKV infection to the TOP (late second trimester) may explain the absence of cerebral anomalies, which could have developed later in gestation. This may explain the negative amniocentesis, which despite having been performed after 21 WG [8], the time lapse between maternal infection and amniocentesis (3 weeks) may have been too short to observe viral excretion into amniotic fluid.

Fetal risks associated with a maternal ZIKV infection remain unclear. Initial reports from Brazilian cohorts suggested rates as high as 46% of adverse outcomes in cases of ZIKV in utero exposure, independent of the timing of exposure [9]. On the other hand, more recent studies have failed to observed similar results [10, 11]. Preliminary data from a recent French Guyana cohort study suggest a much lower rate with 1.7–3% of anomalies observed associated with first or second trimester infection only, despite a transmission rate of 10.9% [12], while rates of 8, 5 and 4% of neurological anomalies (including neural tube defects) have been reported in case of RT-PCR confirmed maternal infection occurring during first, second and third trimesters, respectively [13]. The present work highlights the fact that despite circulation of ZIKV virus, additional factors may contribute to or cause fetal pathology, even in the presence of a confirmed ZIKV fetal infection. An exact diagnosis is mandatory to draw definitive conclusions, but may be difficult to obtain and requires invasive materno-fetal procedures (ie. amniocentesis, fetal blood sampling), as well as advanced microbiological and genetic investigations. Similarly to other congenital infections, it is very likely that not all infected fetuses will become symptomatic. By analogy with CMV, late maternal infection may be associated with pauci- or asymptomatic fetal disease despite higher transmission rates with advancing GA (up to 60–70%) [14]. It is prudent to be reminded that despite the wide distribution of CMV, severe congenital infections are only observed in 0.7% of pregnancies [14]. Although ZIKV harbors some specific characteristics, it seems unlikely that the risk would be significantly higher.

## Abbreviations

BPD: Biparietal diameter
CGH: Comparative genomic hybridization
CMV: Cytomegalovirus
GA: Gestational age
Hb: Hemoglobin
HC: Head circumference
IUGR: Intra-uterine growth restriction
PVB-19: Parvovirus b 19
WG: Weeks gestation
ZIKV: Zika virus
